# Recent Advances in Phytochemical-Based Topical Applications for the Management of Eczema: A Review

**DOI:** 10.3390/ijms25105375

**Published:** 2024-05-15

**Authors:** Janani Radhakrishnan, Barry E. Kennedy, Erin B. Noftall, Carman A. Giacomantonio, H. P. Vasantha Rupasinghe

**Affiliations:** 1 Department of Plant, Food, and Environmental Sciences, Faculty of Agriculture, Dalhousie University, Truro, NS B2N 5E3, Canada; 2 Department of Pathology, Faculty of Medicine, Dalhousie University, Halifax, NS B3H 2Y9, Canada; 3 Department of Surgery, Faculty of Medicine, Dalhousie University, Halifax, NS B3H 2Y9, Canada

**Keywords:** phytochemicals, topical, atopic dermatitis, SCORAD, skin, immune response, nanocarriers

## Abstract

Eczema (atopic dermatitis, AD) is a skin disease characterized by skin barrier dysfunction due to various factors, including genetics, immune system abnormalities, and environmental triggers. Application of emollients and topical drugs such as corticosteroids and calcineurin inhibitors form the mainstay of treatments for this challenging condition. This review aims to summarize the recent advances made in phytochemical-based topical applications to treat AD and the different carriers that are being used. In this review, the clinical efficacy of several plant extracts and bioactive phytochemical compounds in treating AD are discussed. The anti-atopic effects of the herbs are evident through improvements in the Scoring Atopic Dermatitis (SCORAD) index, reduced epidermal thickness, decreased transepidermal water loss, and alleviated itching and dryness in individuals affected by AD as well as in AD mouse models. Histopathological studies and serum analyses conducted in AD mouse models demonstrated a reduction in key inflammatory factors, including thymic stromal lymphopoietin (TSLP), serum immunoglobulin E (IgE), and interleukins (IL). Additionally, there was an observed upregulation of the filaggrin (FLG) gene, which regulates the proteins constituting the stratum corneum, the outermost layer of the epidermis. Carriers play a crucial role in topical drug applications, influencing dose delivery, retention, and bioavailability. This discussion delves into the efficacy of various nanocarriers, including liposomes, ethosomes, nanoemulsions, micelles, nanocrystals, solid-lipid nanoparticles, and polymeric nanoparticles. Consequently, the potential long-term side effects such as atrophy, eruptions, lymphoma, pain, and allergic reactions that are associated with current topical treatments, including emollients, topical corticosteroids, topical calcineurin inhibitors, and crisaborole, can potentially be mitigated through the use of phytochemical-based natural topical treatments.

## 1. Introduction

Eczema is a recurrent inflammatory skin disease characterized by pruritic lesions and dryness of the skin [1]. Eczema adversely affects the overall health of both the affected individual and their families, giving rise to psychosocial difficulties, disturbances in sleep patterns, and sudden episodes of intense itching [2]. The pathophysiology is complex, involving a combination of genetic, immune, and environmental triggers that can result in skin barrier dysfunction and immune abnormalities that often emerge in early childhood [3]. Eczema may be atopic or non-atopic, depending on the presence or absence of immunoglobulin E (IgE) antibodies. The term “atopic” typically denotes IgE sensitization, and this can be identified through the IgE-antibody determination test [4]. Atopic eczema or atopic dermatitis (AD) is the first manifestation of the sequence of IgE responses called the ‘atopic march’, which includes asthma and allergic rhinitis that present in adolescence [1]. According to the International Study of Asthma and Allergies in Childhood (ISAAC), 15–20% of children and 1–3% of adults are affected by eczema [5].

Mainstream treatments, including topical corticosteroids (TCSs) and calcineurin inhibitors (TCIs), are key for symptom management due to their anti-inflammatory and barrier-enhancing effects [6]. However, patient adherence remains a challenge, primarily due to steroid phobia and concerns about long-term TCS usage [7]. Prolonged or inappropriate use of high-potency TCSs can result in cutaneous and systemic adverse events, including skin atrophy, bruising, dyspigmentation, and even serious conditions such as suppression of the hypothalamic–pituitary–adrenal axis, femoral head osteonecrosis, glaucoma, hyperglycemia, and hypertension [7]. Furthermore, the risk of developing a condition known as TCS withdrawal, or TCS-induced rosacea-like dermatitis, adds to the apprehension, as discontinuation of TCS treatment can lead to the exacerbation of AD symptoms [7]. In the case of TCIs, which are commonly used for their non-steroidal approach to treating AD, their side effects include a transient burning sensation, pruritus, and erythema. Additionally, chronic TCI use has been historically associated with widely held yet clinically unfounded concerns regarding increased risks of developing certain skin cancers and even lymphomas. These concerns contribute to the observed low adherence to traditional topical therapies, necessitating the exploration of complementary options. 

Topical agents are preferred when it comes to treating skin diseases, including eczema, for many reasons: the active component of the drug escapes first-pass metabolism, the concentration of the drug in the target tissue is effectively increased, and systemic side effects can be significantly reduced [8]. Pre-clinical studies show that phytochemicals, through their potent antioxidant potential, could emerge as promising candidates for topical treatments [9]. This review aims to consolidate recent advances in phytochemical-based topical agents and elucidate their mechanisms of action in treating AD. 

This review article is based on a literature review of selected relevant published studies published between 2020 and 2024. The databases used included PubMed, Scopus, and Google Scholar. The keywords used in the search included “phytochemicals + eczema”, “topical treatments + eczema”, “herbs + topical + eczema”, “topical + carrier + eczema”, “phytochemicals + dermatitis”, “clinical studies + topical + eczema”, and “nanocarriers + eczema”. Out of over 170 articles collected, only 93 articles were selected using the criteria detailed below. Articles on the oral administration of phytochemicals and the topical application of steroidal creams were excluded. The articles that do not describe the sources of extracts or phytochemicals or details of the experimental model were not included. Articles published before 2020 were considered only in the context of describing the relevant pathophysiology of atopic eczema.

## 2. Pathophysiology of Eczema

**Figure 1:**
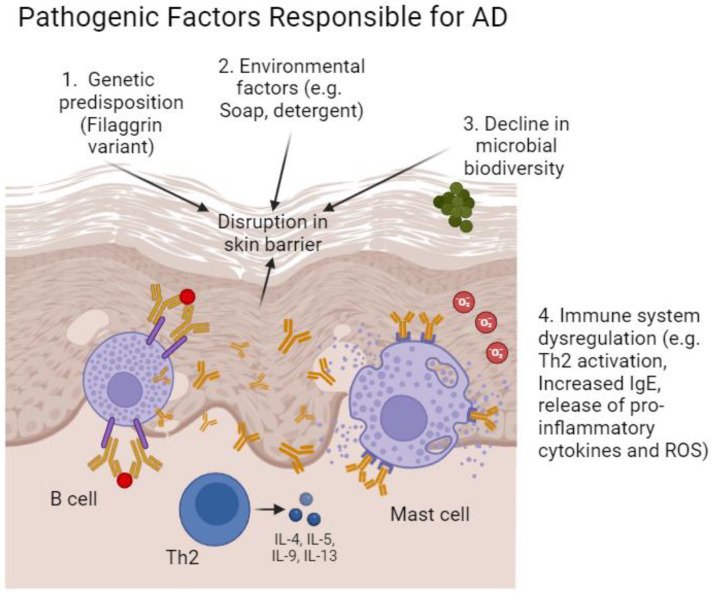
Pathogenic factors contributing to atopic dermatitis. Atopic dermatitis (AD) development is influenced by various factors. These include genetic mutations such as those in the filaggrin gene or other genes associated with skin barrier function. Environmental factors, including cigarette smoke, allergens, pollutants, and chemicals, as well as microbes, can trigger the formation of a factor called thymic stromal lymphopoietin (TSLP). Furthermore, alterations in microbial diversity, particularly an increase in Staphylococcus aureus, are associated with AD progression. Additionally, inflammatory processes play a pivotal role in AD and are characterized by a robust Th2 response, elevated IgE production, increased ROS generation, and mast cell activation. Adapted from [20].

Mutations encoding the filaggrin (filament aggregating protein, FLG) gene are major factors predisposing one to developing atopic eczema (Figure 1). Interestingly, it appears that an FLG mutation in the mother, and not the father, is predictive of the child developing AD even if the child does not inherit the genetic anomaly itself [10]. The FLG gene encodes a structural protein playing a critical role in the terminal differentiation of the epidermis and in skin barrier function [11,12]. 

Various inflammatory immune cells, including macrophages, eosinophils, mast cells, dendritic cells, and T helper type 2 (Th2) cells mediate AD [13]. In AD patients’ skin, inflammation, pruritus, and histamine release occur due to elevated serum IgE concentration resulting from the increased expression of inflammatory cytokines. These typically include interleukins (IL) of the Th2-signaling pathway: IL-4, IL-5, IL-6, IL-10, IL-13, IL-31, and IL-33 [12,14]. Upregulation of IL-4, IL-13, IL-31, and IL-33 downregulates the expression of FLG and other skin barrier-associated genes, including kallikrein, contributing to skin barrier damage. This damage facilitates allergen penetration, reduces skin moisture, and exacerbates inflammation. Additionally, the disruption of anti-microbial peptides leads to allergic inflammation [15]. 

Activation of T lymphocytes induces dendritic cells, eosinophils, mast cells, basophils, and innate lymphoid cells to induce reactive oxygen species (ROS) production, triggering the inflammatory cycle [16]. The intricate interplay among these immune components underscores the systemic nature of AD, which extends beyond localized skin inflammation. A comprehensive understanding of these immunologic intricacies is crucial for advancing personalized diagnostic and therapeutic strategies for patients with AD [13]. 

Environmental factors, including cigarette smoke, allergens, pollutants, chemicals, and microbes, can trigger the formation of a factor called thymic stromal lymphopoietin (TSLP), which is responsible for itching and pruritis [17]. TSLP activates 2-innate lymphoid cells (ILC2), thus elevating Th2 cytokine production in the lesions of AD patients [18]. Skin barrier dysfunction amplifies transepidermal water loss (TEWL) due to decreased ceramides and other water-holding molecules [19]. Moisture loss makes the skin dry, permeable, and susceptible to microbial infection, particularly with *Staphylococcus aureus*, which sticks to the corneocyte of the stratum corneum [20]. *S. aureus* provokes allergic reactions by inhibiting eosinophil apoptosis [21]. The course of AD can lead to the atopic march, which involves the development of asthma and allergic rhinitis [22].

### 2.1. Existing Clinically Proven and Recommended Topical Therapies

Topical corticosteroid therapies are the main line of inflammation management in AD, with systemic corticosteroids used as short-term rescue treatments due to the long-term side effects associated with them [23]. In addition to TCSs, TCIs and crisaborole (a phosphodiesterase inhibitor) are topical treatment agents that are approved by the US Food and Drug Administration (FDA) for AD treatment.

### 2.2. Emollients

Emollients function by forming a protective, occlusive layer on the skin’s surface. This layer serves as a barrier that reduces TEWL by preventing excessive evaporation of moisture from the skin. The occlusive nature of emollients helps to trap water within the epidermis, enhancing skin hydration and contributing to a smoother, more supple skin texture. However, the presence of preservatives and fragrances can cause contact dermatitis and irritation [24].

### 2.3. Moisturizing Products

Reduction of TEWL is important in patients suffering from eczema as it renders the skin dry and increases itching. Moisturizing products such as ceramides and those made from hyaluronic acid-forming saccharide isomerates are used to retain moisture in the stratum corneum [25]. Notably, application of several phytochemicals studied in this review was followed by a reduction in TEWL and an improvement in hydration [26]. 

### 2.4. Topical Corticosteroids

TCSs have been the mainstay of anti-inflammatory treatment for nearly 50 years, as recommended by the American Academy of Dermatology [27]. TCSs act by reducing the effects of pro-inflammatory mediators through steroid receptors in the cells [28]. TCSs also reduce the expression of pro-inflammatory cytokines by impeding antigen processing [29]. Varying potencies of TCSs are prescribed based on vasoconstriction assays [30]. Side effects of long-term use include TCS withdrawal, rosacea-like eruptions, perioral dermatitis, skin atrophy, skin wasting, and in extreme cases, particularly with chronic TCS use, suppression of the hypothalamic–pituitary–adrenal axis and osteoporosis may occur [6,30].

### 2.5. Topical Calcineurin Inhibitors

TCIs (e.g., tacrolimus ointment and pimecrolimus cream) are non-steroidal topical treatments that are recommended for proactive and reactive treatments, having gained FDA approval in 2001 [31]. They reduce the expression of pro-inflammatory cytokines by inhibiting the activation of T lymphocytes [6,31]. Topical tacrolimus and pimecrolimus have anti-pruritic effects and are considered good options for long-term treatment as they do not cause skin atrophy [32]. However, due to the lack of long-term safety data and the theoretical risk of the development of malignancies in patients based on pre-clinical data and post-marketing reports, in 2005 the US FDA introduced a black box warning label for these TCIs. Consequently, there is a hesitancy for prescribers to use them, particularly in children and adolescents [33,34].

### 2.6. Crisaborole

Crisaborole belongs to the class of phosphodiesterase-4 (PDE4) inhibitors and is recommended for reactive therapy, having been FDA-approved in 2016 [30]. PDE4 is a regulator of inflammatory cytokine production [35]. By locally targeting PDE4, crisaborole helps to modulate inflammatory responses in the skin, making it effective in managing certain dermatological conditions. The adverse effects due to long-term application that have been identified include worsening dermatitis, application-site pain, and infection [35]. Careful consideration of these factors is essential when prescribing crisaborole. 

### 2.7. Other Topical Drugs

Difamilast is a non-steroidal and selective topical phosphodiesterase-4 inhibitor that has shown antipruritic and anti-inflammatory properties. Difamilast has been given manufacturing and marketing approval in Japan for the treatment of adult and pediatric patients (2 years of age and older) with AD [36]. The pan-Janus kinase (JAK) inhibitor delgocitinib, in a cream formulation, has been shown to give a dose-dependent response in chronic hand eczema in a randomized phase IIb clinical trial [37]. This drug has also received approval from the Pharmaceuticals and Medical Devices Agency (PMDA) in Japan for the treatment of AD. 

## 3. Classical Animal Models Used to Study Treatments for Eczema

To study the effects of bioactive phytochemicals on eczema symptoms, experimental mouse models that somewhat mimic the clinical symptoms of eczema are frequently employed. Topical agents typically include 2,4-dinitrochlorobenzene (DNCB), a hapten that is widely used to cause a hypersensitivity reaction that is like contact dermatitis by repeated induction because of its ability to effectively penetrate the skin barrier [38]. Although the pathogenicity of contact dermatitis and atopic dermatitis are different, DNCB treatment can result in symptoms like swelling, dryness, and erythema, which are the core of the phenotypic expression of AD [39]. Oxazolone, another haptenation agent, similarly causes atopic dermatitis-like changes in mouse models with increased epidermal and ear thickness [40]. Both DNCB and oxazolone also induce immune responses like the increased secretion of pro-inflammatory and inflammatory cytokines (interleukins and tumor necrosis factor-alpha [TNFα]); increased infiltration of mast cells, eosinophils, lymphocytes; and a spike in serum IgE as are observed in cases of AD [41]. Phthallic anhydride, an industrial chemical, and toluene 2,4-diisocyanate can cause irritant dermatitis by eliciting inflammatory responses and they are also used to induce dermatitis symptoms in mice [42,43]. 

## 4. Plant Extracts and Phytochemicals with Immunomodulatory Effects and Their Potential for Use in the Treatment of Eczema

Several clinical studies have highlighted the therapeutic efficacy of herbal extracts in managing eczema (Table 1). Consequently, exploring phytochemicals capable of inhibiting IgE and cytokines, particularly IL-4, which is a key player in IgE hyperproduction, holds promise for potential interventions [44]. 

Phytochemical-based therapies modulate a broad range of immune and inflammatory pathways to ameliorate various aspects of AD. In addition to reducing TEWL, phytochemicals decrease the infiltration of eosinophils and mast cells, lower serum IgE production, reduce the production of pro-inflammatory cytokines, and inhibit the nuclear factor kappa B (NF-kB) pathway. 

As examples of the spectrum of treatment effects observed after using phytochemicals in experimental models, extracts of Cortex Moutan (*Paeonia suffruticosa* Andrew)*,* Herba Menthae (*Mentha haplocalyx* Briq.), and common marigold (*Calendula officinalis*) reduced production of IL-6, TNFα, IL-8, and monocyte chemoattractant protein-1 (CCL2) in stimulated immune cell lines in vitro, and these agents alleviated ear redness, swelling, and inflammation in oxazolone-induced AD in a mouse model [45]. Similarly, mamiran cream, a Chinese herbal formula, also suppressed histamine release, reduced IgE production, and decreased Th2 responses in DNCB-induced models of AD [46]. Topical application of purified grape polyphenol reduced the epidermal thickness in AD mouse models and exhibited anti-inflammatory effects in a way that was comparable to the commercially used tacrolimus [42]. In other experimental models, an anti-inflammatory effect is attributed to the presence of proanthocyanidins that inhibit the pro-inflammatory cytokines, thus leading to low hyperkeratosis [47]. Topical treatment with *Rosa davurica* Pall prevented adverse systemic immune responses in DNCB-induced AD mice. These included atrophy and decreased white pulp area in the spleen, alongside the suppression of NF-κB and alleviation of erythema [48]. Furthermore, use of the extract derived from *Nymphoides peltata* roots (NPR) led to the effective inhibition of inflammatory cytokines, the reduction of AD-like symptoms, and the enhancement of skin barrier functions. The study also identified three phenolic acids within the NPR extract, namely chlorogenic acid; 3,5-dicaffeoylquinic acid; and 3,4-dicaffeoylquinic acid. Overall, these findings suggest a therapeutic potential for NPR extract in both preventing and treating AD, in addition to highlighting its properties such as anti-inflammatory activity, antioxidant activity, and promoting skin barrier improvement [49].

Targeting the inhibition of TSLP is an important and effective strategy to improve AD symptoms. Mastic, a resin from *Pistacia lentiscus,* reduced the itching effects of AD by reducing TSLP levels in the skin. It also exhibited significant anti-inflammatory properties by downregulating IgE levels, cytokine production, and lymphocyte proliferation in AD mouse models [43]. The multifunctional impacts of mastic positions it as a compelling candidate for continued investigation, thus holding potential implications for the comprehensive management of AD. 

*Chrysanthemum boreale* essential oil and eucalyptus oil contain 1,8-cineole, which suppresses histamine release by regulating SNARE protein (soluble N-ethylmaleimide-sensitive factor activating protein receptor)-associated mast cell degranulation [50]. *Tripterygium wilfordii* Hook, a member of the Celastraceae family of vine-like plants, has long been held as a traditional Chinese medicine for its purported anti-inflammatory and immunosuppressive properties. Celastrol, isolated from *Trypterygium wilfordii* Hook, has been shown to reduce TSLP levels in keratinocytes in addition to decreasing the group 2 innate lymphoid cells (ILC2) population and inactivating NF-κB in a DNCB-induced AD model [51]. Other herbs such as *Lycopus lucidus, Viola yedoensis,* and magnoflorine from *Rhizoma coptidis* have also been found to reduce levels of interleukins and to inhibit the actions of TNFα and NF-κB factors [52,53]. Diosmin, a flavone glycoside found in citrus fruits, plays a unique role in alleviating skin barrier dysfunction by activating aryl hydrocarbon receptors, which regulate the production of skin barrier proteins, including filaggrin and loricrin [54]. 

Furthermore, phytochemicals have shown beneficial effects in human studies. Topical application of marshmallow (*Althaea officinalis*) ointment has demonstrated higher efficacy in children with AD than has topical hydrocortisone [55,56]. The active compounds in marshmallow were found to directly dock to and inhibit IL6, TNFα, and PDE4 isoenzymes through hydrogen bond interactions of hydroxyl groups [55,56]. *S. balsamita* extract, which is rich in flavonoids, exhibits antioxidant and anti-inflammatory activities and decreases the amount of TEWL, thereby restoring the barrier function of the epidermis [26]. Topical application of a herbal cream containing silymarin, which is a polyphenolic flavonolignan from *Silybum marianum* L. (milk thistle) with anti-inflammatory properties, along with an extract from *Fumaria officinalis*, which is a small plant with high antioxidant and immunomodulating activity, significantly improved SCORAD scores in patients [26]. *Indigo naturalis*, an herbal medicine with a historical usage dating back to ancient times, improved EASI within 6 weeks of topical application in AD patients [57]. *I. naturalis* is known to repair abnormal epidermal barriers by promoting differentiation, upregulating claudin-1 expression, restoring tight-junction proteins, and inhibiting keratinocyte proliferation [58,59]. *M. sylvestris*, a medicinal plant used as a remedy for eczema in traditional Persian medicine, gave significant improvement over four weeks in children AD patients upon topical application [60]. Topical application of Olivederma (a combination of *Aloe vera* and virgin olive oil) significantly decreased SCORAD scores and improved quality of life after six weeks in AD patients [61]. This study showed that Olivederma reduced eosinophil and serum levels of IgE [61]. Another clinical study revealed that topical application of an agent known as dwarf elder, which has been historically used as a medication since ancient times, significantly improved itching scores and quality of life to a greater degree than did hydrocortisone cream after 4 weeks of treatment in AD patients [62]. Previous studies have identified anti-inflammatory actions of *S. ebulus* such as the modulation of TNF-α production, IL8 secretion, and activation of the COX-2 pathway mediated by its active compound: ursolic acid [63,64,65]. After 4 weeks of treatment with *Portulaca oleracea* L. (purslane), a medicine traditionally used in Persia for skin ailments, AD patients showed significantly improved fissure scores, participant-reported itching, total itching, and dryness compared to AD patients treated with placebo [66]. Previous studies in DNCB-induced mouse models have demonstrated that purslane lowers serum IgE, mast cell levels, and histamine skin concentration, potentially through its targeting of IFN-γ, TNFα, and IL-4 [67]. Pumpkin pulp is rich in β-carotene, fatty acids, moisture, and flavonoids and has proven that it can be used for many skin disorders such as dermatitis, and that it can be given orally or topically [68]. Application of pumpkin pulp extract as an ointment improved AD patient DLQI scores within four weeks [69]. Collectively, these findings strongly suggest that phytochemicals hold significant potential as effective treatments for AD.

**Table 1:** Plant sources used to prepare and assess phytochemical-rich extracts against eczema.

Plant Source for Extract	Major Active Compound(s)	Experimental Method	Observations and Proposed Mechanisms of Action	References
**(A) Cell models**				
Eucalyptus oil	1,8-cineole	IgE-mediated local allergic cell model.	Suppressed degranulation of mast cells.	[70]
Pure compound	Diosmin	Human skin equivalent model (HSE) and normal human epidermal keratinocytes (NHEK).	Upregulated of skin barrier proteins filaggrin, loricrin, and involucrin by interacting with aryl hydrocarbon receptor (AhR) in NHEK and increased epidermal thickness in HSE.	[54]
**(B) Experimental animal models**				
Acorn shell extract	Gallic acid, ellagic acid	Oxazolone-induced BALB/c mouse model. 2,4-dinitrochlorobenzene (DNCB)-induced AD-like lesions on SKH-1 hairless mice.	Reduced epidermal thickness and infiltration of mast cells. Reduced expression of pro-inflammatory cytokines. Reduced serum IgE and IL-4 production. Improved skin hydration through the reversal of skin barrier dysfunction.	[71]
*Vitis vinifera* seed extract and polyphenolic fraction	Whole grape seed extract and purified polyphenol fraction	60 male albino pthalic anhydride induced AD mice.	Reduced hyperkeratosis.	[42]
Tri-herb formula with *Paeonia suffruticosa, Mentha haplocalyx,* and *Calendula officinalis*	-	Oxazolone-induced AD-like mice model.	Downregulated inflammatory IL-1β, IL-6, IL-8, and TNF-α in human mast cells. Reduced epidermal thickness and mast cell infiltration.	[45]
*Chrysanthemum boreale* essential oil	1,8-cineole [61]	DNCB-induced BALB/c AD mouse model.	Reduced histamine release and increased expression of skin barrier proteins filaggrin and loricrin in keratinocytes.	[50]
*Rhizoma coptidis*	Magnoflorine	DNCB-induced AD mice model.	Decreased caspase-3 expression and inhibited the apoptosis of keratinocytes.	[72]
*Rosa davurica* Pall. Leaves	ND	DNCB-induced AD mice model.	Suppressed serum IgE level and pro-inflammatory cytokines.	[48]
Mastic (resin from *Pistacia lentiscus*)	ND	Histological evidence and toluene-2,4-diisocyanate topical application induced allergic contact dermatitis in NC/Nga mice.	Reduced itch behavior, transepidermal water loss, and skin thickness. Suppressed pro-inflammatory cytokine production and reduced T-cells, and IgE-B cells. Reduced cytokine production in keratinocytes.	[43]
Mamiran cream (stem of *Coptis chinensis*, gall of *Quercus infectoria,* roots of *Rumex dentatus*, and petals of *Rosa rugosa*)	Berberine and gallic acid	DNCB-induced AD mice model.	Improved skin lesions and decreased expression of inflammatory cytokine.	[46]
*Trypterygium wilfordii*	Celastrol, a pentacyclic triterpenoid	House dust mite-stimulated NC/Nga mice.	Reduced levels of TSLP and Th2 cytokines.	[51]
Brown algae	Phlorotannins	Radiation-induced BALB/c dermatitis mouse model.	Activated anti-inflammatory and anti-oxidative stress signaling by augmenting nuclear factor erythroid 2-related factor 2 (Nrf2)/heme oxygenase-1 (HO-1) pathway.	[73]
*Lycopus lucidus*	ND	DNCB-induced Atopic Dermatitis mouse model.	Reduced dermal and epidermal thickness; reduced serum IgE and IL-6 levels; inhibited expression of NF-kB and infiltration of mast cells, eosinophils, and CD8^+^ cells.	[52]
*Viola yedoensis* ethanol extract	ND	DNCB-induced Atopic Dermatitis mouse model.	Decreased hyperkeratosis and infiltration of inflammatory cells; decreased serum IL-6, IL-1β, and tumor necrosis factor-alpha (TNF-α).	[53]
*Fritillariae thunbergia* Bulbus chloroform fraction	ND	DNCB-induced Atopic Dermatitis mouse model.	Reduced epidermal thickness, loss of skin barrier proteins, and infiltration of inflammatory cells.	[74]
**(C) Human studies**				
*Althaea officinalis* flower extract	ND	Double-blind controlled trial phase-II in 40 patients with atopic eczema.	SCORAD was significantly lower, no side effects were observed. Inhibitory interactions with IL6, TNF-alpha, and PDE4.	[55]
*Stizolophus balsamita* extract	Quercetin kaempferol, taxifolin	60 healthy Caucasian adult females.	Improved skin hydration and reduced transepidermal water loss.	[26]
*Fumaria officinalis + Silybum marianum* herbal cream	Isoquinolinic alkaloids and silymarin	Randomized double-blind controlled clinical trial of 40 patients with mild-to-moderate eczema.	Reduced the severity of eczema symptoms indicated by SCORAD Index.	[75]
*Indigo naturalis*	Indirubin	Randomized double-blind clinical trial, 48 participants aged 6 to 65 years with AD affecting less than 40% of their body surface area.	Reduced symptoms as indicated by reduced EASI score. Improved IGA levels, percentage of body surface area with atopic dermatitis involvement, Pruritus VAS, and DLQI/CDLQI score.	[57]
*Malva Sylvestris*	ND	Fifty-one children with AD were randomly enrolled in two arms of a randomized, double- blind, controlled clinical trial.	Reduced symptoms as indicated by skin thickening score, redness score, and SCORAD.	[60]
*Aloe vera* and olive oil *Sambucus ebulus* *Portulaca oleracea* *C. moschata* oil extract (Butternut Squash)	ND ND ND β-carotene, fatty acids ad flavonoids	Thirty-six AD patients Ninety-four patients with hand eczema aged 18–60 years were recruited in two groups. Seventy participants with hand eczema were randomly allocated into the intervention (*n* = 35) and placebo (*n* = 35) groups. Randomized, double-blind trial on 60 patients.	Reduced SCORAD severity score, increased quality of life, reduced DLQI score, improved eosinophil count and serum IgE. Reduced HESCI score, reduced itching score, and improved DLQI score. Resulted in lower physician-reported fissure scores, participant-reported itching, dryness and total itching, and dryness and thickness scores. Significant changes in DLQI scores and HECSI scores, better responses for quality of life. No clinical adverse effects were observed.	[61,62,66,69]

ND, active compounds of the extract were not analyzed or reported; SCORAD, Scoring Atopic Dermatitis Index; HECSI, Hand Eczema Severity Index; EASI, Eczema Area and Severity Index; DLQI, Dermatology Life Quality Index.

## 5. Carriers Used in Topical Applications

**Figure 2:**
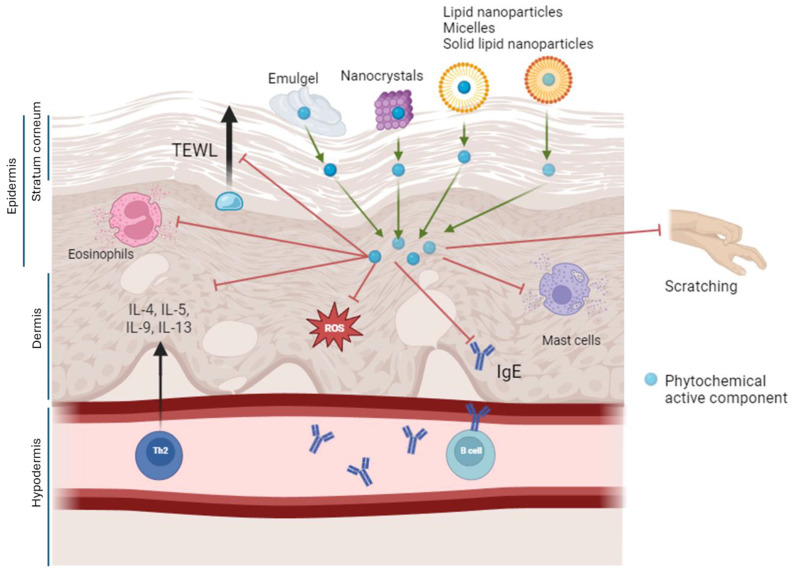
Advanced dermal delivery systems in atopic dermatitis applications. To efficiently deliver the active components of phytochemicals (represented by blue spheres) to both the epidermis and dermis layers of the skin, sophisticated delivery systems capable of overcoming the lipid-enriched corneocytes within the stratum corneum are required. These advanced delivery systems include emulgels, which combine the properties of gels and emulsions; nanocrystals; lipid nanoparticles; micelles; and solid-lipid nanoparticles. Upon successful delivery to the epidermis and dermis, these active compounds can initiate anti-inflammatory actions by targeting various pathways, including the secretion of Th2 cytokines, the production of IgE, the generation of reactive oxygen species (ROS), and the activation and infiltration of eosinophils and mast cells, as well as transepidermal water loss (TEWL) pathways. Cumulatively, these actions contribute to the mitigation of skin barrier damage, reductions in skin dryness, and the alleviation of itching and scratching.

The uppermost layer of the epidermis, the stratum corneum, is composed of 10–20 layers of corneocytes in a lipid-enriched intercellular space and is the primary barrier to the penetration of therapeutic agents [76]. Therefore, the carriers used for dermal delivery are very important. Emulgels, which have the properties of both emulsions and gels, were found to increase the stability and controlled release of the drug compared to creams when the emulgels and creams of *Helichrysum italicum* subsp. *italicum* were tested [77]. Nanotechnological interventions offer advantages in drug delivery and retention due to the size of these carriers. Nano-sized carriers include liposomes, solid-lipid nanoparticles, ethosomes, nanoemulsions, polymeric nanoparticles, micelles, and nanocrystals. They provide better dose control, more targeted delivery, better bioavailability, improved immune response, and fewer adverse effects [78]. The therapeutic potential of ethosomal creams as effective drug delivery systems was demonstrated by experiments in which the tea tree oil-ethosomal creams containing phosphatidylcholine and ethanol exhibited increased penetration of the stratum corneum, increased entrapment efficiency, and more uniform dispersion, together resulting in decreased eosinophil and neutrophil infiltration in BALB/c mice [79]. Similarly, improved delivery of quercetin was observed when nano-lipoidal systems were used. Medical textiles made of poly(vinyl butyral-co-vinyl-alcohol-co vinyl acetate) (PVB) nanofibers have exhibited enhanced oil-delivery performance that improves hydration in atopic skin treatments compared to treatment using textiles made with microfibers [80]. Nano-elastic liposomes (NEL) improve drug delivery by carrying both lipophilic and lipophobic drugs. The NELs are bilayer vesicles that can deliver therapeutic drugs to the dermal layers due to their flexibility that is conferred by the presence of edge activators like sodium cholate and its derivatives [81]. Sodium stibogluconate and ketoconazole co-loaded with NELs were effective against Leishmaniasis [81]. Ultradeformable liposomes (ULs) or transferosomes are nanocarriers that enter the systemic circulation through small tight-junction aqueous channels. Ammonium glycyrrhizinate-loaded ultra-deformable liposomes enabled systemic delivery and exhibited an anti-inflammatory effect on human volunteers. When nano-emulsion gels were used to treat AD, transepidermal water loss was reduced and drug release was improved compared to market-available control delivery methods [82]. Solid-lipid nanoparticles (SLN) are considered superior to other nanocarriers as they exhibit the positive effects of emulsions, liposomes, and polymeric nanoparticles. When coated with positively charged ligands like chitosan, they can improve drug retention in the inflamed area owing to their bio-adhesive properties [83,84]. In vitro assessment of drug permeation and retention of tacrolimus-loaded SLN gel in Franz diffusion cells of rats showed higher permeation and retention compared to those from gel alone [85]. Micelles are a type of self-assembling nanocarrier that, when incorporated into a hydrogel, relieved skin dryness and improved the skin delivery, retention time, adherence, and spreadability of drugs in the case of hydrocortisone-loaded micelles incorporated into a carbopol hydrogel [86]. Nanocrystals, which show improvements as carriers over other nanocarriers, are unique in achieving 100% drug loading, which was demonstrated by electron paramagnetic resonance in dexamethasone, a glucocorticoid used to control inflammation [76]. Figure 2 summarizes the advanced delivery systems as bioactive phytochemical carriers.

Scratching, which is known to worsen AD, disrupts the skin barrier and leads to asteatosis or dry and fissured skin [87,88]. Moisturizers target four interdependent layers of the skin barrier (physical, chemical, microbiologic, and immunologic), support homeostasis, and facilitate the management of AD [89]. Occlusives, humectants, and emollients block the surface of the outermost layer of the epidermis (stratum corneum) and draw water from the dermis into the epidermis in order to strengthen the physical skin barrier. Acidic moisturizers support the chemical skin barrier by providing optimal conditions for enzyme activity, ceramide production, and skin microbiome maintenance. Moisturizers can also support the immunologic skin barrier by lowering its permeability to allergens and sensitizers. Clinical studies show that the volume of moisturizer applied to patients with asteatotic eczema influences the effectiveness of the treatment. In a single-center, randomized, single-blind (evaluator-blind), dose-comparison study with parallel-dose groups, the results suggested that the application of a dose equivalent to one finger-tip unit of the moisturizer twice daily led to faster remission [88]. Over the last two decades, moisturizer manufacturers have progressed in incorporating bioactive natural ingredients, especially phytochemicals, to improve barrier repair and emollient functions [90]. For example, a topical skin moisturizer incorporated with eucalyptus leaf extract improved skin barrier function of AD patients [91]. A clinical study assessing the role of a non-psychoactive cannabinoid-containing moisturizer demonstrated increased hydration of the skin and improved quality of life in AD patients [92]. A randomized, controlled, investigator-blinded study has demonstrated that a moisturizer containing 0.025% licochalcone A, the major chalconoid polyphenol of the root of the *Glycyrrhiza* species, was effective in the treatment of both acute and maintenance phases in mild-to-moderate childhood AD [93].

## 6. Limitations of Investigations

Mouse models are induced with DNCB, oxazolone, or other allergens as a means of triggering allergic responses. Although the phenotypic characteristics of the symptoms in these models are similar to those of AD, these models can not exactly mimic the AD conditions in humans as the pathogenesis of AD is different from contact dermatitis. As there are different types of eczema that vary in pathogenicity, eczema-treatment effects cannot be generalized. The phytochemical profiles of assessed extracts have not been completely characterized. The most abundant phytochemicals may not be the most efficacious active components of an extract. The pharmacokinetics and pharmacodynamics of phytochemicals present in some herbal extracts/products are not comprehensively understood. Therefore, future investigations should aim for the identification of complete phytochemical profiles and the quantification of known phytochemicals within extracts. The present literature also shows the limits of our knowledge of the mechanisms of action of phytochemical extracts and individual components. The toxicity associated with the use of nanocarriers in some herbal products, and the possibility of facing allergic reactions due to nanocarriers, need to be investigated.

## 7. Conclusions

Phytochemical-based topical applications could potentially be complementary medications in the treatment of eczema. They repair skin barrier proteins, reduce transepidermal water loss, downregulate serum IgE levels, inhibit the production of interleukins, inhibit the infiltration of immune cells, and augment Nrf2-antioxidant enzymatic pathways. Bioactive phytochemicals are effective in improving symptoms such as dryness, itching, and the overall SCORAD index. Treatment with these natural ingredients may increase adherence to medications and reduce the adverse side effects associated with the long-term use of corticosteroids. Preparing composite formulations with many herbal phytochemicals to produce a multi-functional cosmetic cream (“cosmeceutical”) that can treat skin barrier dysfunction and exhibit positive immunomodulatory effects would be beneficial. Utilizing safe nanocarriers as efficient delivery systems can improve the effects of treatments and doses required and reduce the burden of eczema.

## Data Availability

Not applicable.
